# Edema, Hyperpigmentation, Induration: 3 Skin Signs Heralding Danger in Patients on Maintenance Hemodialysis

**DOI:** 10.1097/MD.0000000000003121

**Published:** 2016-03-25

**Authors:** Stefan Becker, Stefan Walter, Oliver Witzke, Andreas Körber, Anja Bienholz, Tanja Kottmann, Andreas Kribben, Gernot Kaiser, Anna Mitchell

**Affiliations:** From the Departments of Nephrology (SB, SW, AB, AK, AM), Infectiology (OW), and Dermatology (AK), University Duisburg-Essen, Essen, Germany; Medical Statistics Hamm (TK), Hamm, Germany; and Department of General, Visceral and Transplantation Surgery (GK), University Duisburg-Essen, Essen, Germany.

## Abstract

Skin changes are common in patients on dialysis. This study focused on putative associations of specific skin findings with comorbidities and mortality.

We performed a retrospective analysis of data from 508 patients on maintenance hemodialysis therapy in 7 centers in the German State of North Rhine Westphalia. Data had been collected by interview, from patient files, and from targeted physical examination in an earlier prospective study screening hemodialysis patients for the presence of nephrogenic systemic fibrosis. While on dialysis, patients’ extremities had been examined for any of the following: edematous skin at the lower extremities, hyperpigmentation, induration, and xerosis cutis. Our present data analyses focused on associated mortality and comorbidities.

Five hundred eight patients (median age 71 years, range 20.0–95.9; n = 292 men) had agreed to participate in the initial study: 48% (n = 243) were diabetics and 46% (n = 232) had been diagnosed with coronary heart disease. On examination, 86% of patients (n = 439) presented with at least 1 of the prespecified skin changes. Skin edema (n = 89; 18%), hyperpigmentation (n = 74; 15%), and induration (n = 9; 2%) were independently associated with increased mortality over 24 months (*P* < 0.002, *P* < 0.030, and *P* < 0.020, respectively).

In our study, prespecified skin changes indicated an increased mortality risk in patients on chronic hemodialysis. Routinely assessing the skin of dialysis patients represents a simple, reliable, and cost effective means of identifying those at greatest risk.

## INTRODUCTION

Skin disorders are common in patients with chronic kidney disease (CKD) and are encountered in more than 75% of patients receiving dialysis treatment.^1^ Typical clinical findings include pruritus, xerosis cutis, hyperpigmentation, edematous skin, and calcific uremic arteriolopathy.^1–2^ While these dermal abnormalities are related to the underlying renal disease in some patients, they have also been associated with “uremia in a broad sense.”^3^ We have previously performed a study aimed at detecting patients with nephrogenic systemic fibrosis (NSF).^4^ Now we retrospectively analyzed data collected in this earlier study to better understand skin changes in CKD in the context of their comorbidities.

CKD is a strong risk factor for cardiovascular diseases (CVDs), which may go unnoticed for lack of clear-cut clinical indicators.^5^ We hypothesized that specific skin changes of the extremities, which are easily identifiable on a dialysis ward round, could be indicators of cardiovascular comorbidities. We chose mortality as well as a variety of comorbidities and their associations with skin changes as prespecified main endpoints of this analysis.

## METHODS

### Ethics

Ethics approval was obtained from the ethics committee of University Hospital Essen (08-3912). This study was conducted in accordance with the Declaration of Helsinki.

### Study Design

Data were collected at 7 dialysis centers in the German State of North Rhine Westphalia (Germany) between April 2009 and September 2010. All patients on hemodialysis at these centers were asked to participate (n = 575). Informed consent was a precondition for participation, there were no exclusion criteria; 508 patients participated in the study and were examined while on dialysis prior to gathering the medical history to avoid bias. Data were then collected by interview and from medical records (Table 1). Data on dialysis quality (Kt/V and hemoglobin) were obtained from attending nephrologists. Survival of patients was followed up for a period of 24 months.

**TABLE 1:**
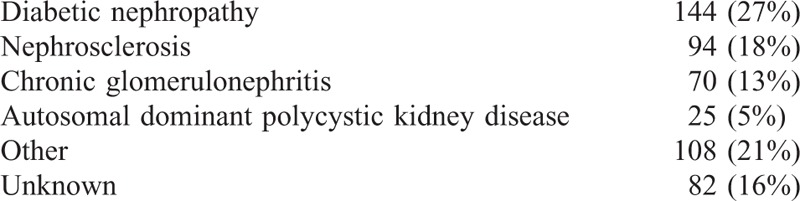
Underlying Kidney Disease (Double Diagnoses in 15 Patients)

### Physical Examination

Patients were evaluated by a trained examiner (SW). One hundred sixty-four patients were independently examined by SB to assess the reproducibility of findings. Reproducibility was 89%. During dialysis, patients’ extremities were examined for any of the following findings^6^.

#### Edematous Skin

Pressure was applied to a small area over the skin. Edema was judged to be present, when an indentation formed which persisted after pressure was released.

#### Indurated Skin

Skin on the extremities was gently palpated in a circular motion with increasing pressure. Induration of the skin was diagnosed, if the skin was found to have lost elasticity and pliability.

#### Hyperpigmented Skin

Skin appearance of the lower extremities was compared to that of skin elsewhere because hyperpigmentation of the extremities is a common clinical sign in patients with NSF. Hyperpigmentation was diagnosed, if the skin was darker than elsewhere.

#### Xerosis Cutis

Xerosis cutis was diagnosed, if the skin appeared to be excessively dry to the eye and to touch.

All skin changes were assessed categorically.

### Statistical Analysis

Statistical analysis was performed using SPSS 19 (IBM Corporation, Armonk, New York, United States). Data are presented as descriptive statistics; categorical variables are expressed as percentage. For comparisons of categorical variables in those who died and in survivors, such as the concurrence of edematous skin with or without coronary heart disease chi-squared test was employed. In addition to the univariate analysis, we performed multivariate stepwise logistic regression analyses. These included age, sex, diabetes, hypertension, coronary heart disease, history of stroke, autoimmune disease, liver disease, and history of thrombosis as well as the skin findings to evaluate the associations of skin changes and comorbidities. A multivariate analysis model assessing the associations of skin changes and mortality included age, sex, edema, hyperpigmentation, induration, and xerosis cutis both with and without Kt/V. *P* values <0.05 were considered statistically significant.

## RESULTS

Five hundred eight patients (median age 71, range 20.0–95.9 years) participated in the study. Of these, 292 (58%) were men. Overall, patients had been on maintenance hemodialysis for a median of 2.9 years (range 1 day to 33.5 years). In all patients, biocompatible membranes were used for dialysis. Blood count hemoglobin concentrations >10 mg/dL were present in 91% of patients. Data for Kt/V were supplied for 492 patients. Kt/V was >1.2 in 419 individuals (85%).

Diabetic nephropathy was the most prevalent underlying kidney disease, followed by nephrosclerosis (Table 1).

The prevalence of the comorbidities assessed and their influence on survival is shown in Table 2. Age older than the median of 71 years, diabetes mellitus, coronary heart disease, and peripheral arterial disease were more prevalent in individuals who died over 24 months.

**TABLE 2:**
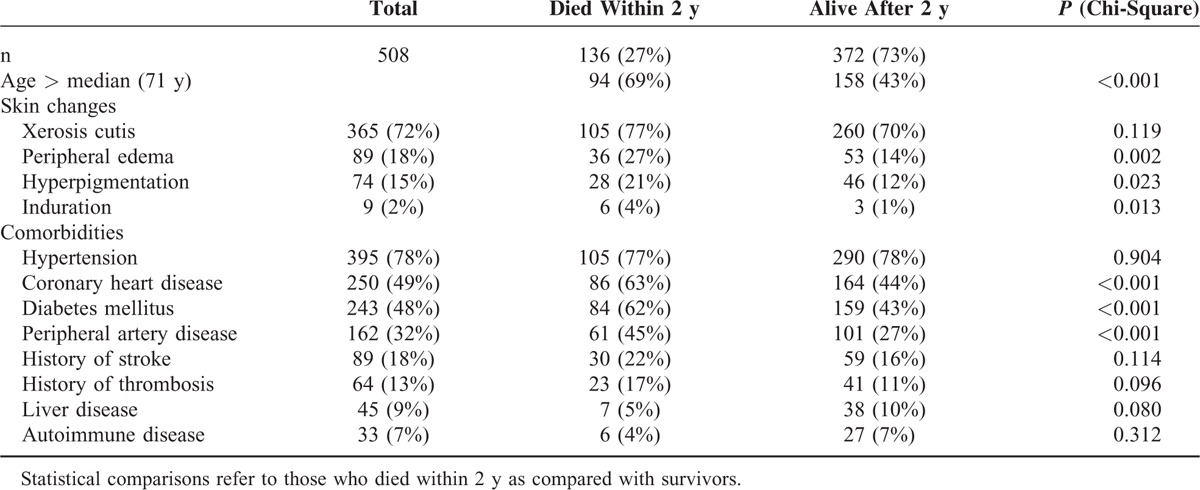
Clinical Findings, Comorbidities, and Associated Mortality

Four hundred thirty-nine patients (86%) presented with at least 1 of the prespecified skin changes. Xerosis cutis was the most prevalent skin finding, followed by edema, hyperpigmentation, and induration (Table 2).

In Kaplan–Meier survival analyses edema, hyperpigmentation, and induration were associated with increased mortality (log-rank; *P* < 0.002, *P* < 0.030, and *P* < 0.020, respectively).

**FIGURE 1:**
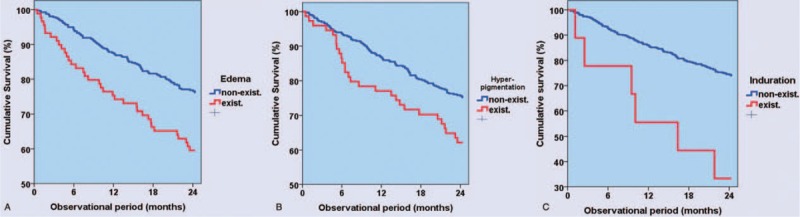
(A–C) Association of specific skin findings with mortality. In the course of the 24 mo observational period, edematous skin changes (A: log-rank; *P* < 0.002); hyperpigmentation (B: log-rank; *P* < 0.030); and induration (C: log-rank; *P* < 0.020) were associated with reduced survival.

In the univariate analysis, presence of skin edema showed the strongest association with death after 2 years, followed by hyperpigmentation, each with a near double prevalence in those who died. Induration although diagnosed in only 2% of patients was also associated with death, while dry skin was diagnosed at a similar rate in survivors and in those who died (Table 2). Figure 1A–C shows survival curves for patients presenting with and without edema, hyperpigmentation, and induration, respectively.

Edema was associated with a history of thrombosis and coronary heart disease, while hyperpigmentation was associated with peripheral arterial disease and liver disease (Tables 3 and 4). Edema and hyperpigmentation were also associated with each other.

**TABLE 3:**
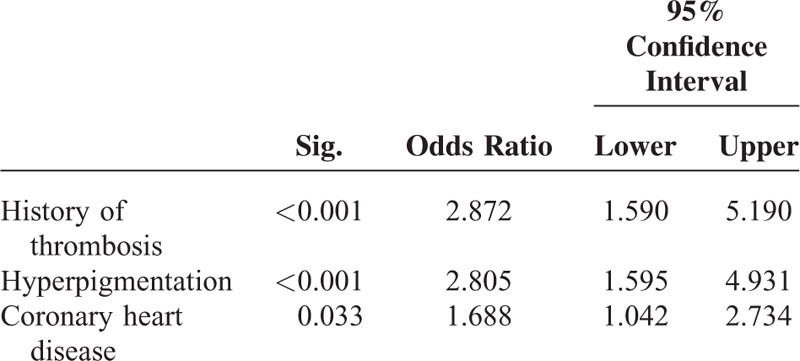
Comorbidities/Skin Changes Independently Associated With Edematous Skin

**TABLE 4:**
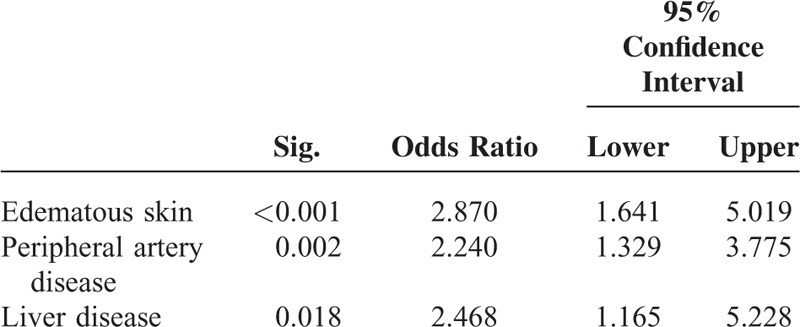
Comorbidities/Skin Changes Independently Associated With Hyperpigmentation

In the multivariate stepwise logistic regression analyses, age, induration, and edema were independently associated with mortality, irrespective of whether Kt/V was introduced into the model (Table 5).

**TABLE 5:**
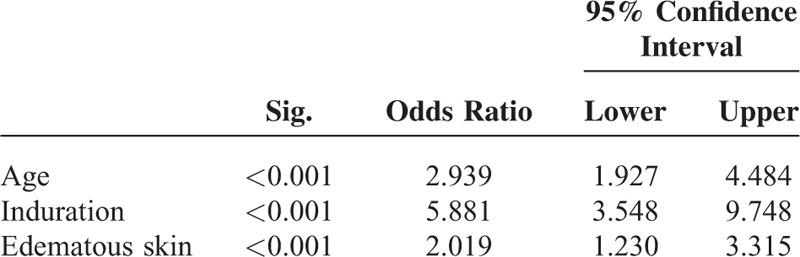
Comorbidities/Skin Changes Independently Associated With Mortality

Dry skin showed a strong association with diabetes but was also associated with liver disease and with edema (Table 6). It was not associated with an increased mortality risk.

**TABLE 6:**
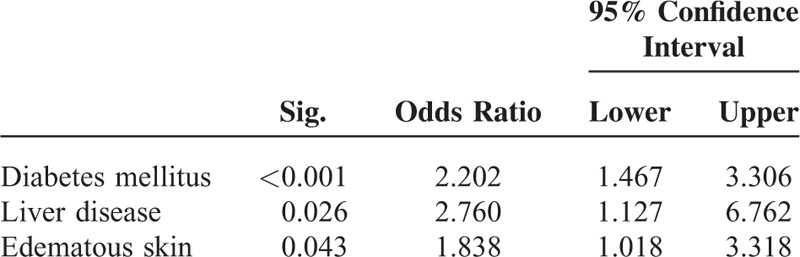
Comorbidities/Skin Changes Independently Associated With Xerosis Cutis

## DISCUSSION

Changes of skin morphology and function frequently occur in patients suffering from CKD and may seriously affect quality of life.^3,7^ The present study is the first to describe that common skin affectations may serve as visible indicators of CVD and an increased mortality risk in patients on chronic hemodialysis.

## REVIEW OF FINDINGS

### General

While traditional risk factors are important predictors of CVD in individuals with CKD, they do not fully reflect the CVD burden in these patients: risk scores such as the Framingham equations have been shown to be insufficient instruments for assessing morbidity and mortality risk in CKD.^8,9^ Following the implementation of a staging system for kidney disease, it has become evident that, while CVD risk is highest in patients on dialysis, it is increased at all stages of impaired kidney function. Concurrently a variety of so-called “nonclassical” risk factors associated with a higher incidence of cardiovascular events in CKD, that is, abnormal mineral metabolism, anemia, and chronic inflammation, were identified.^8^ While the relevance and value of such risk factors mainly assessed by laboratory measurements remains undisputed, our data indicate that the prognostic significance of specific clinical and therefore readily accessible indicators for CVD in CKD may still be underestimated. In our study, edema and induration of the skin on the lower extremities were associated with an increased mortality risk in the univariate and survival analyses. Comorbidities frequently occurring with CKD stage 5D are arterial hypertension, diabetes mellitus, and the various manifestations of CVD—coronary heart disease and central or peripheral artery disease. Our patient cohort reflects this. Epidemiological data on survival of patients on hemodialysis vary. Mortality risk is highest in diabetics and in old patients, which is also mirrored in our cohort. The fact that 25% of the patients who participated in this study died within 2 years must be regarded as deeply disturbing. It demands meticulous consideration of all contributing factors in order to identify measures that can be taken to reduce mortality risk and possibly enhance quality of life.

### Edematous Skin

Edema is a common clinical sign and the list of illnesses associated with edema formation is long.^10^ The mechanisms underlying edema development and their interplay are complex and remain to be fully identified. The physiological forces involved include the net difference of intracapillary and extracapillary hydrostatic pressure, differences in oncotic pressure between blood vessels and the interstitium, and the permeability of the blood vessel wall. Importantly, mechanisms of interstitial water and solute handling, and, more specifically, removal of solutes and water from the skin via the lymph system have to be taken into account.^11^ From clinical experience edema in patients on hemodialysis frequently signify volume overload. However, heart failure, chronic liver disease, and chronic venous insufficiency can cause edema even in the absence of impaired kidney function. In these instances, “third-spacing” of fluid is secondary to hydrostatic or osmotic forces and may well be accompanied by a reduced circulatory blood volume. In our patients, edematous skin was most strongly associated with a history of thrombosis, with hyperpigmentation and with coronary heart disease. If only speculatively we would like to offer a hypothesis on the underlying pathophysiology: chronic inflammation is the common denominator of CKD and all comorbidities assessed in this study. It is tempting to speculate that where edema in hemodialysis patients cannot directly be attributed to volume overload, they may be the consequence of the chronic inflammation associated with end-stage renal disease. The dysregulation of the immune system described for CKD at its various stages promotes vascular dysfunction, that is, arterial capillary leakiness. In this instance, an excess of fluid, proteins, cells, and electrolytes may leave the arterial vasculature and promote interstitial inflammatory responses. Edema form, if the finely tuned mechanisms on the level of the endothelium are disturbed, that regulate venous re-uptake and function on the one hand and lymph vessel formation, and lymph-generation and -transport on the other. While endothelial dysfunction in blood and lymph vessels secondary to chronic inflammation may suffice to explain local or general skin edema; this will likely be compounded in the presence of volume overload. In the setting of a dialysis ward, it is mandatory to differentiate between the various causes of edema, if a correct decision is to be made on which postdialysis weight should be aimed for in each patient. Here clinicians tread a fine line: in order to prevent intra or postdialytic hypotension that would predispose to myocardial stunning and its deleterious consequences, the goal for postdialytic weight may be set higher rather than lower. A recent publication advocates permissive volume overload as a safety measure.^12^ However, there is accumulating evidence that volume overload significantly increases mortality risk and should be avoided.^13,14^ Since ours is a retrospective analysis we cannot definitely conclude, which were the underlying causes in the patients presenting with edema. Our data add to the findings condemning volume overload in chronic hemodialysis. Additionally, though, they suggest that edema, an easily accessible clinical sign, may be a significant surrogate for cardiovascular and mortality risk irrespective of their underlying pathophysiology.

### Hyperpigmentation

Hyperpigmentation has been described to be present in more than 30% of patients with estimated glomerular filtration rate <15 mL/min.^15^ Its prevalence was comparatively low in our cohort overall; however, 1 in 5 of those who died had hyperpigmented skin areas on the extremities. It has been suggested that urochrome pigments, carotenoids, and melanocyte-stimulating hormone may be responsible for the increased pigmentation observed in CKD.^16,17^ Of these, the effects of melatonin may be the most significant: recent data stress its role as a scavenger of reactive oxygen species (ROS) in the skin and elsewhere.^18^ Again speculatively, the tanned aspect of the skin in some hemodialysis patients could therefore reflect an enhanced melatonin action in response to higher oxidative stress. In the same line, bilirubin has also been identified as an ROS scavenger.^19^ Thus, while the association of hyperpigmentation with liver disease in our study may be secondary to reduced glucuronidation, it could also denote a counter regulatory mechanism in the presence of oxidative stress. Others have described both positive and negative correlations of bilirubin and kidney function.^20^ The association of hyperpigmentation with peripheral artery disease suggests a role for trophic disturbances secondary to low blood flow resulting in hypoxia and inflammation.

### Induration

Induration of the skin is a hallmark of NSF, which was the reason, why this criterion was chosen when data were collected for our initial study on NSF prevalence in patients on hemodialysis.^4^ NSF occurrence has been intimately linked to reduced kidney function and use of contrast agents containing gadolinium (GBCAs) for magnetic resonance imaging. Edema and proinflammatory conditions have been reported among the additional risk factors.^21^ A histological diagnosis of NSF was made in skin biopsies of 2 of the 9 patients with induration of the skin described here. Clinical features of NSF resemble those of systemic sclerosis.^22,23^ In both of these diseases, dysregulated matrix remodeling and signs of inflammation have been reported together with skin fibrosis.^22,23^ However, while systemic sclerosis has been identified as a vascular disease with pulmonary hypertension being the leading cause of death, death of patients with NSF has mainly been linked to fibrosis of internal organ structures.^24^ NSF has recently also been reported in patients who did not receive GBCAs.^25^ This opens the possibility that we may not have identified all instances of NSF, because at the time of our initial study skin biopsies were only offered to the 2 patients with indurated skin who had received GBCAs. Although the incidence of NSF seems to have decreased with the use of less toxic GBCAs, to date it has been shown to occur as late as 8 years after the last GBCA application^26^ and own observation. In the absence of histological assessments in the 7 remaining patients and presuming, they did not suffer from “classical” NSF we can only speculate on their tissue changes. It would seem feasible that chronic volume overload irrespective of its cause leads to interstitial hypertension. Elevated pressure in the interstitium alone or together with other factors such as an increased interstitial load of proteins, lipids, and electrolytes may promote inflammation, structural matrix changes, and fibrogenesis. In euvolemic patients, chronic inflammation as a sequelae of kidney disease or CVD alone may trigger altered matrix formation: sodium seems to be 1 of the triggers of chronic inflammatory responses. Recently excess storage of sodium has been detected in the skin of hemodialysis patients.^27^ Investigators from the same group have described migration of cells from the mononuclear phagocyte system to the skin interstitium in response to high sodium and have been able to link these responses to alterations in lymph vessel formation and to aggravation of salt sensitive hypertension in experimental animals.^28^

### Xerosis Cutis

Dry skin is frequently encountered in CKD patients (50% to 85%).^29,30^ Its mean prevalence of 72% in our cohort is in keeping with those earlier results. Xerosis cutis is caused by atrophy of sweat- and sebaceous-glands and disturbed dermal hydration, at times attributed to the use of diuretics.^17^ Additionally autonomous neuropathy as a hallmark of diabetes, uremia, and liver disease^31,32^ may contribute to xerosis cutis. Dry skin was similarly prevalent in patients who died and in those who survived, which may explain why it was not associated with an increased mortality risk.

### Limitations

Although the overall sample size is small, to our knowledge our study is by far the largest study to date that systematically evaluated skin changes in patients on hemodialysis and the only study that assesses the relationship of skin changes with comorbidities and mortality. The sample size was determined by the fact that this study is a retrospective analysis from an earlier, prospective investigation on the prevalence and incidence of NSF in an unselected patient cohort. The prevalence of diabetes and coronary artery disease (CAD) was high in our population (48% and 49%, respectively). However, it can safely be assumed that this reflects characteristics of patients on hemodialysis in Germany as well as in the United States. In Germany, the initiative “Qualität in der Nephrologie” (QiN) has analyzed data from 18,000 dialysis patients treated at the German centers of the “Kuratorium für Dialyse und Nierentransplantation e.V.” (KfH). Thus far unpublished data show a prevalence of 43.4% for diabetes and of 37.2% for coronary heart disease in incident dialysis patients in 2011.^33^ A similar diabetes prevalence for German patients on dialysis has since been documented in the Dialysis Outcomes and Practice Patterns Study Program DOPPS.^34^ Incidence of both diabetes and coronary heart disease rises with age and with time on dialysis. If 1 takes into account that our study did not only assess patients new to dialysis treatment, it is likely that the prevalence of diabetes and CAD in our study is typical of German hemodialysis patients. Finally it would have been interesting to see, if and how the additional information generated by body composition analysis would have added to our main results. Future studies should make use of such technology.

### Interpretation

In our study, prespecified skin changes indicated an increased mortality risk in patients on chronic hemodialysis. From our results we hypothesize that persistent hypervolemia and chronic inflammation alone or together trigger a pathophysiological sequence, in which chronic peripheral edema precede hyperpigmentation and induration of the skin. We thus create a new pathophysiological concept linking skin changes in dialysis patients with diseases of the internal organs.

Physicians in developed countries may be reminded by our data that clinical observation and a hands-on approach can substantially add to the information gained from laboratory values or technical procedures. Routinely assessing the skin of dialysis patients represents a simple, reliable, and cost effective means of identifying those at greatest risk. While this is useful in industrialized countries it may be invaluable in countries where medical resources are limited. In all settings, patients profit from the personal care that comes with the touch of a hand.
